# Viral vector‐based gene therapies in the clinic

**DOI:** 10.1002/btm2.10258

**Published:** 2021-10-20

**Authors:** Zongmin Zhao, Aaron C. Anselmo, Samir Mitragotri

**Affiliations:** ^1^ Department of Pharmaceutical Sciences, College of Pharmacy University of Illinois at Chicago Chicago Illinois USA; ^2^ Division of Pharmacoengineering and Molecular Pharmaceutics, Eshelman School of Pharmacy University of North Carolina at Chapel Hill Chapel Hill North Carolina USA; ^3^ John A. Paulson School of Engineering and Applied Sciences Harvard University Cambridge Massachusetts USA; ^4^ Wyss Institute for Biologically Inspired Engineering Harvard University Boston Massachusetts USA

**Keywords:** adeno‐associated virus, adenovirus, clinical translation, clinical trials, gene, gene therapy, gene transfer, herpes simplex virus, viral vector

## Abstract

Gene therapies are currently one of the most investigated therapeutic modalities in both the preclinical and clinical settings and have shown promise in treating a diverse spectrum of diseases. Gene therapies aim at introducing a gene material in target cells and represent a promising approach to cure diseases that were thought to be incurable by conventional modalities. In many cases, a gene therapy requires a vector to deliver gene therapeutics into target cells; viral vectors are among the most widely studied vectors owing to their distinguished advantages such as outstanding transduction efficiency. With decades of development, viral vector‐based gene therapies have achieved promising clinical outcomes with many products approved for treating a range of diseases including cancer, infectious diseases and monogenic diseases. In addition, a number of active clinical trials are underway to further expand their therapeutic potential. In this review, we highlight the diversity of viral vectors, review approved products, and discuss the current clinical landscape of in vivo viral vector‐based gene therapies. We have reviewed 13 approved products and their clinical applications. We have also analyzed more than 200 active trials based on various viral vectors and discussed their respective therapeutic applications. Moreover, we provide a critical analysis of the major translational challenges for in vivo viral vector‐based gene therapies and discuss possible strategies to address the same.

## 1 INTRODUCTION

Gene therapy, modifying expression of genes or correcting dysfunctional genes, offers great potential as a therapeutic modality for treating a plethora of diseases.^1^, ^2^, ^3^ Unlike traditional drugs, gene therapy genetically modifies cells and thus opens possibilities of curing diseases that were once thought to be incurable.^4^, ^5^ The concept of gene therapy can date back to the 1960s when early studies demonstrated that DNA sequences could be introduced into mammalian cells for gene repair.^6^ Decades of scientific efforts led to the first human gene therapy clinical trial in 1990 using a retrovirus vector technology for treating severe combined immunodeficiency.^7^, ^8^ However, gene therapy experienced a major setback in late 1990s following the death of a patient due to immune responses caused by the viral vector in a trial in 1999 and the development of viral vector‐induced leukemia in four patients after receiving a retrovirus‐based gene therapy in another trial in 2000.^9^ These two events brought gene therapy to a temporary halt in the clinic, raised concerns about its safety, and highlighted the critical need for safer viral vectors. The following decade focused on better understanding of the biology of viral vectors and advancing the engineering of safe and effective vectors; this led to the first clinical approvals of gene therapy products. China approved the first gene therapy in the world for treating head and neck cancer in 2003 (Gendicine). EMA approved its first gene therapy product in 2012 (Glybera), and the United States approved its first in 2017 (Kymriah).^10^ With the development of gene editing technologies (e.g., CRISPR/Cas9) that could precisely modify genes at a base level,^11^, ^12^, ^13^ gene therapy is stepping into a new era and rapidly expanding its therapeutic horizon in treating a broader spectrum of diseases.

**FIGURE 1:**
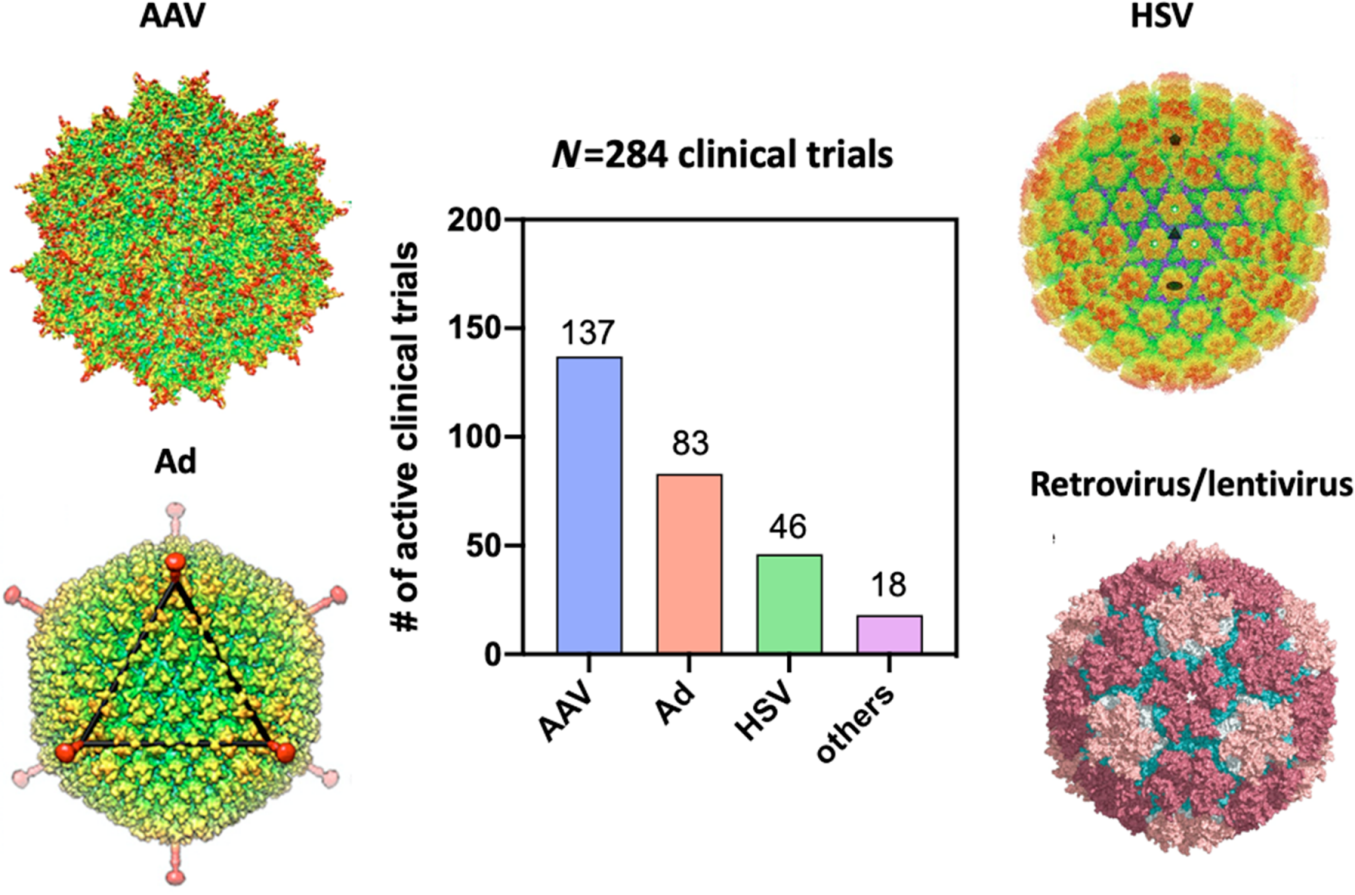
Major clinically relevant viral vectors and active clinical trials analyzed in this review. Structure of representative viral capsids (AAV, Ad, HSV and retrovirus) reconstructed from Cryo‐EM was presented. Images of structure of viral capsids were adapted/reprinted from the following literature: adeno‐associated virus, adapted from Reference 20; Ad, reprinted from Reference 21; HSV, reprinted from Reference 22 with permission from the American Association for the Advancement of Science; retrovirus, reprinted from Reference 23

Current gene therapies fall into two broad categories, that is ex vivo and in vivo gene therapies. Particularly, in vivo gene therapies involve the direct infusion of gene therapeutics into patients' bloodstream or injection into target organs.^12^, ^14^ A vector is generally required for in vivo gene therapies, with an aim to pack and deliver gene therapeutics into target cells. Engineered viruses are the dominant vectors in current gene therapy clinical studies.^15^, ^16^ To date, a plethora of viral vectors including adenovirus (Ad), adeno‐associated virus (AAV), and herpes simplex virus (HSV) have proven their potential in safely and efficiently delivering gene therapies.^17^, ^18^ Excitingly, over a dozen viral gene therapy products have been approved for treating cancer, infectious diseases, and monogenic diseases and their clinical investigation is still expanding.^5^ Here, we provide an overview of clinical advances of viral vector‐based gene therapies. Particularly, we restrict our discussion to in vivo gene therapies. Clinical advances of ex vivo gene therapies, often related to a subclass of cell therapies, have been recently discussed in other reviews.^19^ We identified more than 10 approved products and more than 200 active clinical trials (Figure 1) and provided a snapchat of the current clinical landscape by discussing the diversity of clinically relevant viral vectors, reviewing the clinical applications of approved products, and discussing active clinical trials based on their corresponding vectors and disease indications. We also discuss critical challenges associated with the clinical translation of in vivo viral vector‐based gene therapies, with an aim to provide a framework for better product design.

## 2 CLINICALLY RELEVANT VIRAL VECTORS

The primary reason of using a virus as a vector for gene delivery is to employ their natural capability of infecting cells and to efficiently shuttle gene materials of interest into host cells.^17^ Major viral vector types used for in vivo gene therapies in the clinic include Ad, AAV, HSV, retrovirus, and lentivirus (Figure 1).^24^, ^25^ Here we briefly introduce these vectors with a focus on their primary applications and distinguishing characteristics. Comprehensive reviews of different viral vectors can be found elsewhere.^18^, ^24^

Adenovirus (Ad) is one of the earliest viral vectors investigated in the clinic for in vivo gene therapies.^26^, ^27^, ^28^ Ads are a class of DNA viruses with a 34–43 kb genome enclosed in a nonenveloped icosahedral viral particle.^29^ There are more than 50 Ad serotypes, with Ad5 and Ad26 being most widely used for gene therapy.^16^ Three generations of Ad vectors have been engineered to fit different therapeutic applications. The first generation involves the removal of the E1 and E3 units that makes the vector capable of carrying up to 7.5 kb foreign DNA.^30^, ^31^ The E1 and E4 units are deleted in the second generation of Ad vectors that significantly reduces immunogenicity.^32^ All viral genes are deleted in the third generation that allows the vector to carry more than 30 kb foreign DNA.^33^ Notable characteristics of Ad vectors include: (1) multiple genome copies can be delivered into one host cell that can lead to a high gene expression level; (2) gene expression by Ad vectors is transient because DNA cargo is episomal and not integrated into host genome; (3) they can transduce both dividing and nondiving cells; and (4) they are highly immunogenic.^34^ Because of these characteristics, Ad vector‐based gene therapies are primarily utilized for treating cancer and vaccination against infectious diseases.^35^, ^36^

Adeno‐associated virus (AAV) is the most widely used viral vector for in vivo gene therapy applications. AAV is a nonpathogenic parvovirus with a 4.7 kb DNA genome enclosed in a nonenveloped icosahedral capsid.^37^ AAV has 11 natural serotypes and over 100 variants.^38^, ^39^ Different serotypes have tropism toward different tissues that makes each serotype suitable for gene delivery to specific tissues.^40^ For example, AAV9 exhibits tropism toward central nervous system (CNS) organs while AAV8 can effectively transduce pancreas.^18^, ^41^ Major characteristics of AAV vectors include: (1) they can transduce both diving and nondiving cells and do not integrate DNA into host genome; (2) they can enable long‐term, stable gene expression; (3) they have low immunogenicity. Due to these unique features, AAV is the most suitable viral vector for in vivo gene therapies, especially for conditions that require long‐term gene modifications.^42^ Their primary clinical applications cover the treatment of a broad array of monogenic diseases including ophthalmological diseases, metabolic diseases, hematological diseases, neurological diseases, and musculoskeletal diseases.^43^

Herpes simplex virus (HSV) is an enveloped virus with a double‐stranded NDA genome of over 150 kb.^44^ The virus genome encodes approximately 90 genes; half of the genes are nonessential that can be removed/replaced in recombinant vectors and thus affords a high capacity for foreign DNA.^16^ Eight human HSV serotypes have been identified and each serotype exhibits distinct tropism.^18^, ^45^, ^46^ Currently, three major types of HSV vectors have been engineered for gene therapy applications, including amplicon HSV, replication‐defective HSV, and replication‐competent HSV.^16^, ^44^ Amplicon HSV is an engineered vector to carry a large foreign DNA of over 100 kb. Replication‐defective HSV vector is typically created by depletion of necessary genes for the lytic cycle of HSV and is less toxic and immunogenic. Replication‐competent HSV vector is an engineered HSV that keeps the genes for replication in vitro but deletes the genes for replication in vivo.^16^, ^44^ Major characteristics of HSV vectors include (1) their ability to evade the immune system, (2) capability to deliver large DNA cargos and multiple genes, and (3) intrinsic or engineered cell‐specific lytic property.^44^ Clinical application of HSV vector‐based gene therapies has been primarily focused on cancer treatment, mostly attributed to their intrinsic oncolytic capability.^47^

Retrovirus is the first viral vector that was studied in clinical trials for in vivo gene therapy. Retrovirus is an enveloped spherical virus that carries their genetic materials in the form of RNA. Retrovirus vectors can reverse transcribe their genetic materials (single‐stranded RNA) into double‐stranded DNA and integrate it into host cells' genome.^48^, ^49^ Major advantages of retrovirus vectors for gene therapy is that they can carry a large gene of interest (9–12 kb) and result in long‐term gene expression due to their integration into host genome.^50^ However, several major drawbacks limit their applications.^51^ First, retrovirus vectors require cell division to integrate its DNA into host genome, and thus they can only transduce dividing cells. In addition, retrovirus vectors have the risk to randomly insert its DNA into host chromosome and leads to insertional mutagenesis.^51^ Self‐inactivating vectors that have the promotor or enhancer of the long terminal repeat deleted have been developed to reduce the risk of insertional mutagenesis.^16^ Due to these limitations, retrovirus vectors are currently not often used in clinical studies any more.

Lentivirus is another important viral vector for gene therapy. Its major application is for ex vivo gene therapies.^52^ However, it is also currently investigated in the clinic for in vivo gene therapy applications. Lentivirus is a subtype of retrovirus and carries the genetic materials in the form of RNA. However, unlike retrovirus, lentivirus can integrate its genome into and transduce nondividing cells.^53^ The first generation of lentivirus vectors is originally derived from (human immunodeficiency virus 1) HIV‐1 and has proven its capability of efficiently transducing CNS organs in vivo.^53^ New generations of lentivirus vectors are derived from nonhuman lentiviruses and are theoretically more acceptable because their parental viruses are not infectious to humans.^54^ Distinguishing characteristics of lentivirus vectors include: (1) capability of transducing both dividing and nondividing cells, (2) capability to enable long‐term gene expression, and (3) reduced risk of genotoxicity and insertional mutagenesis as compared to retrovirus vectors. The main disadvantage of lentivirus vectors is their limited genetic cargo capability.^16^ The primary application of lentivirus vectors for in vivo gene therapies is to treat monogenic diseases and chronic diseases including neurological diseases, ophthalmological diseases, and metabolic diseases.

Apart from the aforementioned, other viral vectors are also studied in the clinic for in vivo gene delivery, albeit to a lesser extent. These include vesicular stomatitis virus (VSV), modified vaccinia virus Ankara (MVA), arenavirus, Sendai virus, measles virus, to name a few.^55^, ^56^, ^57^, ^58^, ^59^ Of note, clinical application of these vectors is based on their unique properties. For example, VSV is studied for treating liver cancer and advanced solid tumor because of their intrinsic oncolytic capability.^55^, ^60^ MVA is widely exploited for vaccination applications attributed to their tropism toward antigen presenting cells.^61^

## 3 CLINICALLY APPROVED VIRAL GENE THERAPIES

Our search revealed 13 approved in vivo viral gene therapy products globally. These products use HSV, retrovirus, Ad, VSV, MVA, and AAV viral vectors (Table 1). Their clinical use covers therapies of cancer, infectious diseases, and monogenic diseases. In this section, we discuss these approved products and their respective clinical indications.

**TABLE 1:** Clinically approved in vivo viral gene therapies, grouped by broad indications

Name/trade name (manufacturer)	Viral vector type	Approved indications	Approval year	Key outcomes of late‐stage trials leading to approval	Administration routes	Investigated indications
Cancer						
Talimogene laherparepvec/IMLYGIC® (Amgen)	HSV1	Local recurrent unresectable cutaneous, subcutaneous, and nodal melanoma after initial surgery	2015 (USFDA), 2015 (EMA)	Significantly higher response rate (16.3% vs. 2.1%) and improved overall survival (23.3 months vs. 18.9 months) as compared to the control therapy GM‐CSF	Intralesional	Various cancers
Mx‐dnG1/Rexin‐G® (Epeius Biotechnolgies)	Retrovirus	Soft tissue sarcoma, osteosarcoma and pancreatic cancer	2007 (BFAD)	Well‐tolerated and safe; elevated survival rate of patients receiving Rexin‐G® as compared to chemotherapy alone	Intravenous	Breast cancer, osteosarcoma, sarcoma, pancreatic cancer, colorectal neoplasms, COVID‐19
H101/Oncorine® (Shanghai Sunway Biotech)	Ad5	Nasopharyngeal cancer	2005 (NMPA)	Significantly higher overall response rate when in combination with chemotherapy as compared to chemotherapy alone	Intravenous	Refractory malignant ascites, hepatocellular carcinoma
Ad‐p53/Gendicine® (Shenzhen SiBiono GeneTech)	Ad5	Head and neck cancer	2003 (NMPA)	90% total response rate that was significantly higher than that achieved by conventional chemotherapy alone.	Intratumoral, intracavity, intravenous	Various cancers
Infectious diseases (vaccination)						
rVSV‐ZEBOV/Ervebo® (Merck)	VSV	Ebola virus infection	2019 (EMA), 2019 (USFDA)	Vaccine efficacy was 100%; 90.0%–97.8% (1 month after vaccination) and 83.2%–95.4% (6 months after vaccination) of subjects showed antibody response; vaccine was well‐tolerated	Intramuscular	Ebola virus infection
Ad26.ZEBOV and MVA‐BN‐Filo/Zabdeno® and Mvabea® (Johnson & Johnson)	Ad26 and MVA	Ebola virus infection	2020 (EMA)	98%–100% of study participants mounted antibody response after two vaccine doses; vaccine was well‐tolerated	Intramuscular	Ebola virus infection
JNJ‐78436735, formerly Ad26.COV2.S (Johnson and Johnson)	Ad26	COVID‐19	2021 (Approved or authorized for emergency use in >30 countries)	66% overall efficacy for one‐dose vaccination; well‐tolerated	Intramuscular	COVID‐19
Sputnik V, formerly Gam‐COVID‐Vac (Gamaleya Research Institute, Acellena Contract Drug Research and Development)	Ad26, Ad5	COVID‐19	2021 (Approved or authorized for emergency use in >60 countries)	79% overall efficacy for two‐dose vaccination; well‐tolerated	Intramuscular	COVID‐19
Convidicea, aka Ad5‐nCoV (CanSino Biologics)	Ad5	COVID‐19	2021 (NMPA, Mexico, Pakistan, Chile, Hungary, Moldova)	65% overall efficacy for single‐dose vaccination; well‐tolerated	Intramuscular	COVID‐19
AZD1222, aka Covishield in India (AstraZeneca/Oxford)	ChAd	COVID‐19	2020, 2021 (Authorized for emergency use in >110 countries)	76% overall efficacy for two‐dose vaccination; well‐tolerated	Intramuscular	COVID‐19
Ophthalmological diseases						
Voretigene neparvovec/LUXTURNA® (Spark Therapeutics)	AAV2	Leber's congenital amaurosis (Biallelic RPE65 mutation‐associated retinal dystrophy)	2017 (USFDA), 2020 (Health Canada), 2020 (TGA)	Significant improvement in functional vision as compared to control groups, as determined by the multi‐luminance mobility test (MLMT) score change from baseline to Year 1	Subretinal	Leber's congenital amaurosis
Neurological diseases						
Onasemnogene abeparvovec/ZOLGENSMA® (AveXis, now Novartis Gene Therapies)	AAV9	Spinal muscular atrophy (SMA) with bi‐allelic mutations in the survival motor neuron 1 (SMN1) gene in pediatric patients less than 2 years of age	2019 (USFDA), 2020 (EMA), 2020 (JMHW)	Patients treated with ZOLGENSMA® demonstrated significant improvement in their ability to reach developmental motor milestones (e.g., head control and the ability to sit without support)	Intravenous	Spinal muscular atrophy (SMA)
Metabolic diseases						
Alipogene tiparvovec/Glybera® (UniQure)	AAV1	Lipoprotein lipase deficiency	2012 (EMA)	Demonstrated improvement of postprandial chylomicron metabolism, long‐term expression of LPL gene and presence of active LPL protein, decreased trend in incidence and severity of pancreatitis	Intramuscular	Lipoprotein lipase deficiency, Familial hyperchylomicronemia

Abbreviations: Viral vector types: AAV, Adeno‐associated virus; Ad26, Adenovirus serotype 26; Ad5, Adenovirus serotype 5; ChAd, Chimpanzee Adenovirus; MVA, Modified vaccinia virus Ankara; HSV, Herpes simplex virus; VSV, Vesicular stomatitis virus. Agencies: BFAD, Bureau of Food and Drug, aka Philippine FDA; EMA, European Medicines Agency; JMHW, Japanese Ministry of Health and Welfare; NMPA, National Medical Products Administration, formerly China Food and Drug Administration (CFDA); TGA, Therapeutic Goods Administration, aka Australian FDA; USFDA, The United States Food and Drug Administration.

### 3.1 Cancer therapy products

Four viral gene therapy products have been approved globally for the treatment of cancer, solid tumors in particular (Table 1). Two of them are based on adenovirus and the remaining two use HSV or retrovirus as viral vectors. The principal mechanism of action of these products is based on: (1) the engineered viral vectors have intrinsic oncolytic properties and/or (2) the carried gene leads to expression of tumor suppressors or immunomodulators that enhance the anti‐tumor efficacy.^18^ One notion is that following their approval for a specific cancer type, all of these products have been or are being studied in the clinic for treating various additional types of cancers.

IMLYGIC® is the first and only US FDA‐approved viral gene therapy for cancer. IMLYGIC® was approved for the treatment of local recurrent unresectable cutaneous, subcutaneous, and nodal melanoma after initial surgery via direct intralesional administration.^62^ IMLYGIC® is based on a modified HSV1 in which two viral genes, γ34.5 and α47, that encode Infected cell protein 34.5 (ICP34.5) and ICP47, respectively, were deleted and replaced with the human granulocyte‐macrophage colony‐stimulating factor (GM‐CSF) gene.^63^ Depletion of the γ34.5 gene enables the virus to selectively replicate in tumors but not in normal tissues. Deletion of the α47 gene revokes the suppression of immune responses to the virus and helps activate the immune system. Locally expressed GM‐CSF attracts the infiltration of dendritic cells for tumor antigen presentation and subsequently leads to an adaptive immune response against the tumor.^63^ Of note, apart from melanoma, IMLYGIC® has been clinically investigated for treating other solid tumors.^64^, ^65^ Our search on Clinicaltrials.gov indicated that IMLYGIC® was mentioned in more than 30 active trials studying its capability of treating various cancers such as soft tissue sarcoma, triple negative breast cancer, ovarian cancer, pancreatic cancer, rectal cancer, among others.

Apart from IMLYGIC®, three other cancer viral gene therapy products have been approved by regulatory agencies other than US FDA/European Medicines Agency (EMA) including Gendicine®, Oncorine®, and Rexin‐G®. Gendicine® is the world's first approved cancer viral gene therapy and was approved in China in 2003 for treating head and neck squamous cell carcinoma.^66^ Gendicine® is based on a human Ad5 in which the E1 gene was replaced by the gene encoding human wide‐type p53 that is a tumor suppressor. Distinct from IMLYGIC®, the mechanism of action of Gendicine® depends on the expression of p53 protein in tumor cells that initiates apoptotic pathways, suppresses anti‐apoptotic events, and blocks the survival pathways.^5^ Gendicine® has been clinically studied for treating various cancers such as bladder cancer, ovarian cancer, lung cancer, breast cancer, liver cancer, among others.^67^ It is currently mentioned in an active trial investigating its combination with immune checkpoint inhibitors for treating solid tumors. Oncorine®, another Ad5‐based cancer viral gene therapy was approved in China in 2005 for the treatment of late‐stage refractory nasopharyngeal cancer in combination with chemotherapy. Oncorine® is an oncolytic viral gene therapy and is based on a modified Ad5 in which the viral gene, E1B‐55KD, is completely deleted; no exogenous genes were incorporated into the virus. Defect of E1B‐55KD allows the virus to selectively replicate in p53‐deficint tumor cells but not in normal cells. Virus lyses infected tumor cells and releases from lysed cells to infect neighboring cells.^68^ Oncorine® is currently investigated in active clinical trials for treating additional cancer types including refractory malignant ascites and hepatocellular carcinoma.

Rexin‐G® is a retrovirus‐based cancer viral gene therapy carrying a cytocidal cyclin G1 gene and was approved in the Philippines in 2007 for treating solid tumors. Different from the other three approved products, Rexin‐G® is a tumor targeting viral gene therapy.^69^ Its tumor targeting ability is attributed to the display of a cryotic *SIG*‐binding peptide on the viral vector that can selectively bind to abnormal Signature (*SIG*) proteins in tumors. The mechanism of action of Rexin‐G® is primarily based on the expression of cyclin G1 in tumor cells that arrests the cell cycle in G1 phase and thus triggers cell death and apoptosis.^5^ Following its approval in Philippines, Rexin‐G® has been investigated in several completed Phase 1 or 2 trials in the United States for treating various cancers including pancreatic cancer, sarcoma, breast cancer, and osteosarcoma.^69^, ^70^, ^71^ US FDA granted Rexin‐G® orphan drug designation for osteosarcoma and soft tissue sarcoma in 2008 and fast‐track designation for pancreatic cancer in 2009. Rexin‐G® is currently mentioned in two active clinical trials. Interestingly, one active trial is investigating Rexin‐G® for treating COVID‐19 with the rationale that it can target to exposed collagenous proteins in injured lungs, enter and kill rapidly dividing T cells, and thus reduce cytokine release and acute respiratory distress syndrome.

### 3.2 Vaccination products against infectious diseases

Six viral gene therapy products have been approved or authorized for emergency use for vaccination against infectious diseases, specifically Ebola virus infections and COVID‐19 (Table 1). All of the approved products are based on recombinant replication‐incompetent viral vectors carrying the gene encoding target virus surface proteins. The use of replication‐incompetent viral vectors reduces the safety risks caused by the viral vectors. The mechanism of action of these products depends on: (1) efficient entry of viral vectors into cells at/around injection sites and deliver the gene construct into infected cells and (2) the delivered gene instructing the cells to overexpress target virus surface proteins that stimulate the immune system to induce cellular and humoral responses against the infectious viruses. Notably, all six vaccines, except for JNJ‐78436735 and Convidicea, adopt a two‐dose schedule. Of note, in two products (Zabdeno®/Mvabea® and Sputnik V), two different viral vectors were used in the first and second dose. The underlying rationale seems to be that antibodies against the viral vectors generated after the first dose might neutralize and thus reduce the efficacy of the second dose; the use of a different viral vector in the second dose can bypass this concern.

Two viral gene therapy products have been approved for vaccination against Ebola virus infections. Ervebo® is the world's first Ebola virus vaccine and was approved by EMA and US FDA in 2019. Ervebo® uses recombinant VSV as the viral vector that carries a gene encoding the envelope glycoprotein from the Ebola virus (Zaire strain).^72^ It is currently mentioned in two active trials in Clinicaltrials.gov to study its efficacy in specific population groups or to compare its efficacy to other Ebola vaccines. Different from Ervebo®, Zabdeno®/Mvabea® uses two different viral vectors (Ad26 and MVA) carrying gene constructs encoding the Ebola virus surface glycoprotein.^73^ Our search revealed that nine active trials are associated with Zabdeno®/Mvabea® to further investigate its use for preventing Ebola virus infections.

Four viral gene therapy products have been approved for vaccination against SARS‐CoV‐2 infections. All four vaccines are based on recombinant Ad vectors harboring the gene encoding spike protein S. The rationale underlying the use of Ad may be derived from the rapid but transient gene expression property of the Ad vector.^74^, ^75^ Rapid gene expression enables the quick induction of an immune response while the transient expression nature reduces safety risks. JNJ‐78436735 is currently the only viral COVID‐19 vaccine that has received Emergency Use Authorization in the United States. Unlike other approved COVID‐19 vaccines, JNJ‐78436735 was proven to be safe and effective with just one dose rather than two.^76^ It is currently mentioned in six active trials on Clinicaltrials.gov to study its safety and efficacy in specific population groups including pregnant women and children. The other three approved viral COVID‐19 vaccines adopt similar Ad viral vector‐based design as JNJ‐78436735 and each of them has received approval or authorization for emergency use in different countries. Noticeably, AZD1222, a chimpanzee Ad‐based COVID‐19 vaccine,^77^ was the world's first approved viral COVID‐19 vaccine and has now received authorization for use in more than 30 countries. However, it was recently suspended in several countries due to increased risk of blood clots.

### 3.3 Products for monogenetic diseases

Another important category of approved viral gene therapies is for treating monogenic diseases. In this category, three products have been approved for the therapy of metabolic diseases, ophthalmological diseases, and neurological diseases (Table 1). Although they are indicated for different diseases, their fundamental mechanism of action is similar, that is to deliver a corrected gene to replace the mutated/dysfunctional copy in target cells to alleviate or even cure diseases.^5^ Notably, all three approved products use AAV as the viral vector. This is mostly attributed to the distinct feature of AAVs that they lack pathogenicity, exhibit low immunogenicity, and can typically lead to long‐lasting gene expression.

Glybera® is the world's first approved viral gene therapy for treating a monogenic disease. It was approved by EMA in 2012 for the treatment of lipoprotein lipase deficiency, a metabolic genetic disorder in which a person has a defective gene for lipoprotein lipase. Glybera® uses AAV1 as a vector to deliver an intact copy of the human lipoprotein lipase gene into muscle cells after intramuscular administration.^78^, ^79^, ^80^ Glybera® is currently mentioned in an active clinical trial to study its long‐term safety and efficacy. As the first US FDA approved gene therapy for monogenic diseases, LUXTURNA® received approval in 2017 in the United States and subsequently in Canada and Australia in 2020 for treating Biallelic RPE65 mutation‐associated retinal dystrophy. It is based on an AAV2 vector containing human RPE65 cDNA.^81^ LUXTURNA® is currently in five active clinical trials on Clinicaltrails.gov to investigate its long‐term safety/efficacy or its therapeutic potential in specific population groups. ZOLGENSMA® is the only approved viral gene therapy for treating neurological disorders. It was approved by US FDA in 2019 for treating spinal muscular atrophy with bi‐allelic mutations in the survival motor neurons 1 gene in pediatric patients younger than 2 years of age.^5^ Different from the other two approved products for monogenic diseases, ZOLGENSMA® is injected intravenously. This mostly relies on the intrinsic tropism of the engineered AAV9 vector toward the CNS organ. ZOLGENSMA® is currently mentioned in five active clinical trials to study its long‐term safety and efficacy or to explore its therapeutic potential using other administration routes.

## 4 CURRENT CLINICAL TRIALS FOR VIRAL VECTOR‐BASED IN VIVO GENE THERAPIES

We performed the search for active clinical trials on clinicaltrials.gov using the following methods/criteria. We used “gene therapy” as the keyword in the “Other terms” category and the system also automatically searched for “gene transfer” and “DNA therapy”. Under the “Status‐Recruitment” category, we included the following status: not yet recruiting, recruiting, enrolling by invitation, and active/not recruiting. We then manually went through all the identified trials and excluded nonviral vector‐based gene therapy trials, ex vivo gene therapy trials, and trials of observational studies (rather than interventional studies). The collected data are as of March 2021.

**FIGURE 2:**
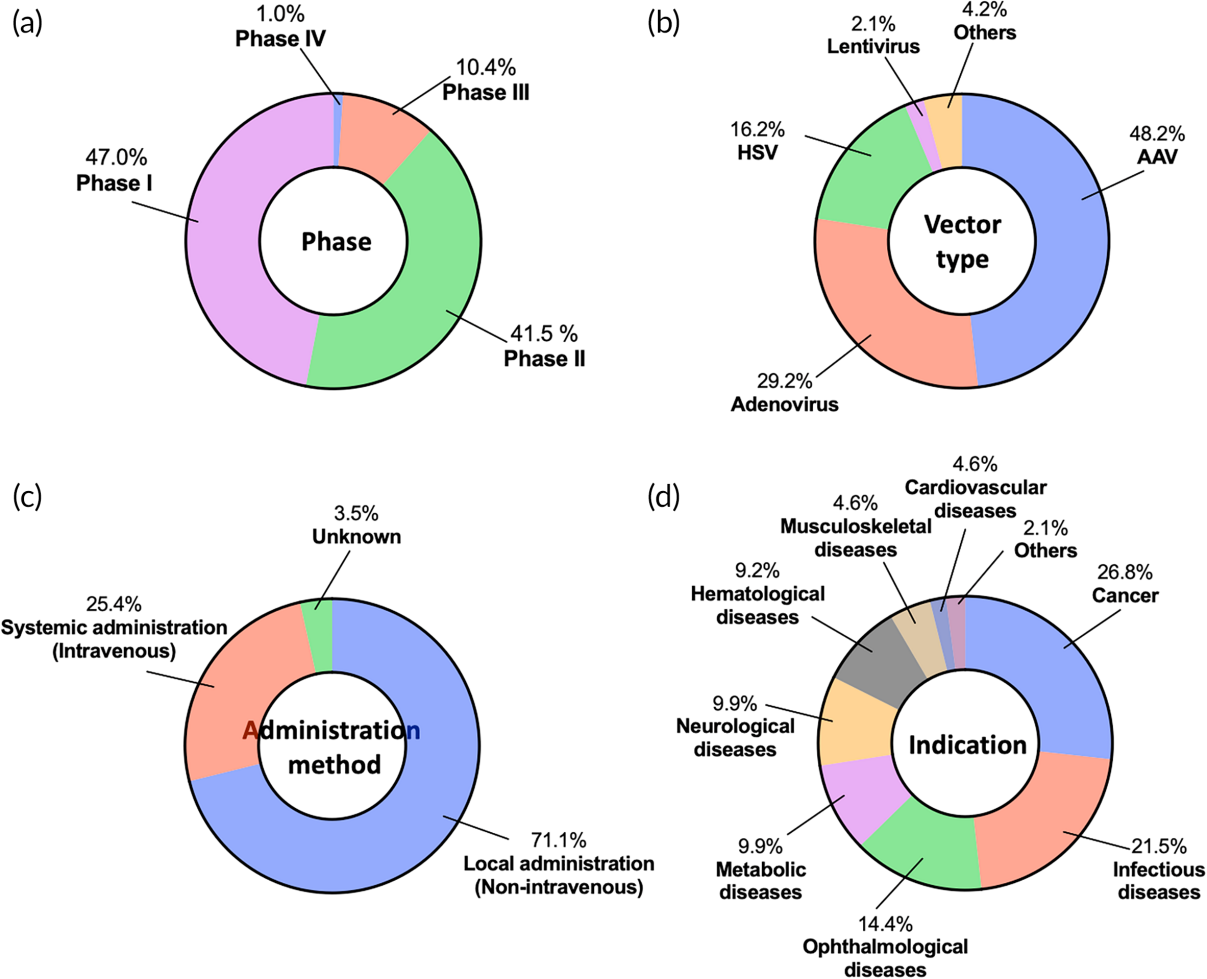
Clinical landscape of viral vector‐based in vivo gene therapies. Overview of current clinical trials based on (a) phase, (b) vector type, (c) administration method, and (d) indication

Our search identified 284 active clinical trials for viral vector‐based in vivo gene therapies. More than 10 vector types are currently used in active trials, among which, AAV, Ad, and HSV are the most used. Particularly, AAV are dominantly utilized, accounting for 48.2% of the identified trials (Figure 2a). This can be most likely attributed to the recent advances in the engineering of various AAV serotypes/variants and their outstanding advantages such as low immunogenicity and stable gene expressions.^37^, ^42^, ^82^, ^83^, ^84^ Of note, Ad and HSV, two of the earliest vectors studied in the clinic, remain frequently utilized in active trials. However, retrovirus is not often used any more (only one active trial identified), most likely attributed to its high risk of insertional mutagenesis.^85^, ^86^ Analysis of the stage of current active trials revealed that majority of the trials are in early stages (Phases 1 and 2). However, more than 10.4% of the trials have reached late stages (Phases 3 and 4) (Figure 2b). Distribution of the phase of the identified trials is indicative of their outstanding clinical potential; many products have been proven safe and reached late‐stage investigations while many more products entered early‐stage trials. In a majority (71.1%) of the identified trials, a non‐intravenous method (e.g., intramuscular, intratumoral, subretinal, intravitreal, intracranial, intrathecal, and subcutaneous) was used to deliver the gene therapy while 25.4% of them were administered intravenously (Figure 2c). Current active trials are investigated for a broad spectrum of indications, which can be classified into three major categories including cancer, infectious diseases, and monogenic diseases (Figure 2d). The cancer trials cover different types of cancers, however, most of them are for treating solid tumors, most likely attributed to the fact that viral vectors can be more effectively delivered to and retained in solid tumors as compared to liquid tumors. Most trials for treating infectious diseases employ a vaccination mechanism. Monogenic disease trials cover a diverse spectrum of indications including ophthalmological, metabolic, neurological, hematological, musculoskeletal, and cardiovascular diseases. Here, we further analyze the identified trials based on the viral vector types and their respective indications.

### 4.1 AAV vector‐based clinical trials

**FIGURE 3:**
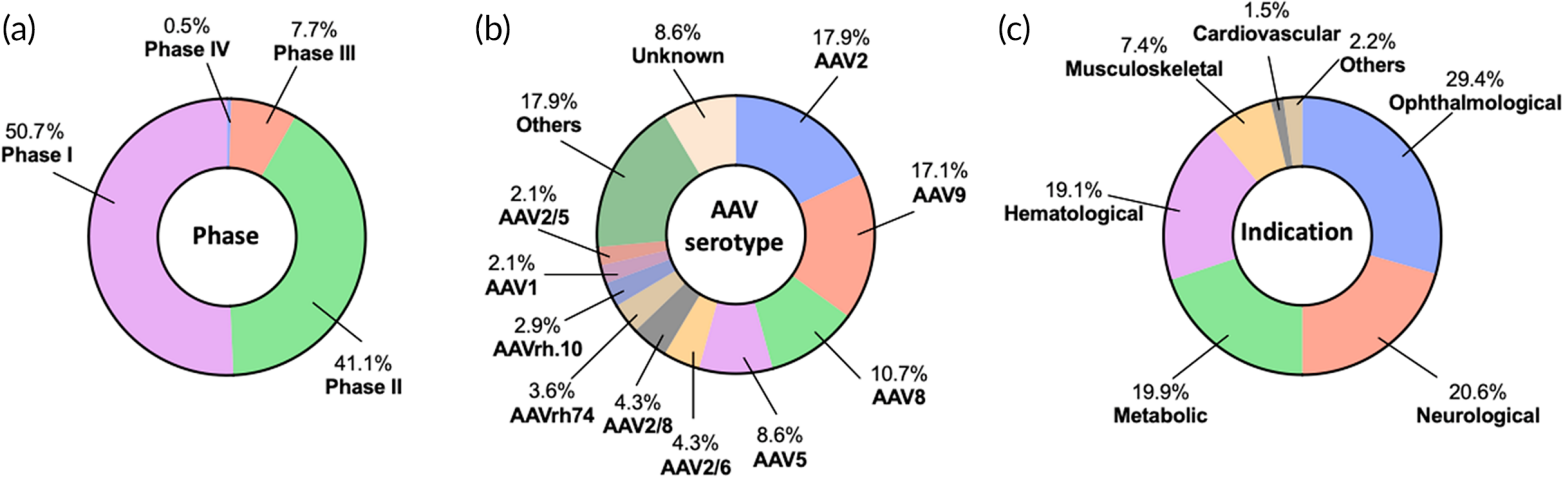
Landscape of adeno‐associated virus‐based in vivo gene therapy clinical trials. A total of 137 active clinical trials were identified and further analyzed according to (a) phase, (b) serotype, and (c) indication

AAV is the most dominant vector type used in the identified trials for in vivo gene therapies. We identified 137 AAV‐based active clinical trials. Majority of them are in early stages (Phases 1 and 2), however, more than 7% of them have reached at least Phase 3 (Figure 3a), reflective of its outstanding clinical potential. Further analysis revealed that the late‐stage trials mainly focus on hematological diseases such as hemophilia A and B, neurological diseases such as spinal muscular atrophy, ophthalmological diseases such as retinitis pigmentosa, and musculoskeletal disorders such as Duchenne muscular dystrophy. We analyzed the serotypes/variants of AAV vectors used in active trials. Historically, AAV2 was the first AAV serotype that was studied in the clinic for in vivo gene therapy.^43^ However, the past decades have witnessed the engineering of a diverse array of AAV serotypes/variants for specific tissue targeting and indications.^42^, ^84^, ^87^ The efforts of engineering serotypes/variants are reflected in the ongoing clinical trials. More than 20 serotypes/variants are used in the current clinical trials, with AAV2, AAV9, and AAV8 being the most widely used (Figure 3b). Long‐studied serotypes such as AAV2, AAV8, and AAV5 have proven their outstanding safety profiles as they have entered Phase 3 trials. New variants such as AAV4D‐R100, AAV4D‐R102, AAV2tYF, AAVS3, AAV.rh8, AAV.rh10h, AAV.rh74, AAVhu37, AAV‐LK03, AAV Spark 10, and some others, are emerging; however, their safety is still being evaluated in early‐stage trials. We analyzed the administration method used in the identified AAV trials. About half (48.2%) of the trials used intravenous injection (Figure S1a). In the context of intravenous administrations, AAV vectors are entrapped in the liver or targeted to CNS organs relying on tropism of specific AAV variants. In the case of non‐intravenous administration, a more localized administration method including subretinal, intracranial, intrathecal, or intravitreal injections is utilized to better localize AAV vectors to target tissues. Analysis of tissue targets of the identified trials suggests that liver, eye, and CNS organs are the most targeted organs; a substantial percentage of trials (8%) also involve muscles as the primary target tissue (Figure S1b).

Monogenic diseases are the major indications the active AAV‐based clinical trials are focused on (Figure 3c and Table 2). This is not surprising because AAV vectors can transduce both dividing and nondividing cells and result in long‐term, stable gene expression, which makes it most suitable for indications that require long‐term gene modifications.^42^, ^88^ Ophthalmological, neurological, metabolic, and hematological diseases are the top disease categories among the identified AAV‐based trials.

**TABLE 2:** Examples of current clinical trials for adeno‐associated virus (AAV)‐based in vivo gene therapies, grouped by indication

Viral vector	Serotype	Gene of interest	Indication	Name (Sponsor)	Route	Trial number
Ophthalmological diseases (*n* = 40)						
AAV	AAV4D‐R100	RPGR	Retinitis pigmentosa (X‐Linked)	4D‐125 (4D Molecular Therapeutics)	Intravitreal	NCT04517149 (Phase 1/2)
AAV	AAV2	sCD59	Age‐related macular degeneration (Dry)	AAVCAGsCD59 (Hemera Biosciences)	Intravitreal	NCT04358471 (Phase 2)
AAV	AAV2	ND4	Leber hereditary optic neuropathy	GS010, aka Lenadogene Nolparvovec (GenSight Biologics)	Intravitreal	NCT03406104 (Phase 3)
AAV	AAV2	REP1	Choroideremia	BIIB‐111 (NightstaRx Ltd)	Subretinal	NCT03507686 (Phase 2)
AAV	AAV2tYF	CNGA3	Achromatopsia	AGTC‐402 (Applied Genetic Technologies Corp)	Subretinal	NCT02935517 (Phase 1/2)
AAV	AAV2	hRPE65	Leber congenital amaurosis	Voretigene Neparvovec (Novartis)	Subretinal	NCT04516369 (Phase 3)
AAV	AAV2/8	hCYP4V2	Bietti's crystalline dystrophy	rAAV2/8‐hCYP4V2 (Beijing Tongren Hospital)	Subretinal	NCT04722107 (Phase 1)
AAV	AAV8	Anti‐VEGF fab	Diabetic retinopathy	RGX‐314 (Regenxbio Inc.)	Intrasuprachoroidal	NCT04567550 (Phase 2)
AAV	AAV2tYF	hRS1	X‐linked retinoschisis	rAAV2tYF‐CB‐hRS1 (Applied Genetic Technologies Corp)	Subretinal	NCT02416622 (Phase 1/2)
Neurological diseases (*n* = 28)						
AAV	AAV9	hSMN	Spinal muscular atrophy	Onasemnogene Abeparvovec‐xioi (Novartis)	Intravenous	NCT03505099 (Phase 3)
AAV	AAV9	GBA1	Parkinson's disease	PR001A (Prevail Therapeutics)	Intracranial	NCT04127578 (Phase 1/2)
AAV	AAV2	AADC	Aromatic L‐amino acid decarboxylase (AADC) deficiency	AAV2‐Haadc (University of California, San Francisco)	Intracerebral	NCT02852213 (Phase 1)
AAV	AAVrh.10 h	hAPOE2	Alzheimer disease	AAVrh.10 hPOE2 (Weill Medical College of Cornell University)	Intracranial	NCT03634007 (Phase 1)
AAV	AAVrh.10	hGALC	Krabbe disease	FBX‐101 (Forge Biologics, Inc)	Intravenous	NCT04693598 (Phase 1/2)
AAV	AAVrh.10	GLB1	GM1 gangliosidosis	LYS‐GM101 (LYSOGENE)	Intravenous	NCT04273269 (Phase 1/2)
AAV	AAV1	GRN	Frontotemporal dementia	PBFT02 (Passage Bio, Inc.)	Intra cisterna magna	NCT04747431 (Phase 1/2)
AAV	AAV1	NTF3	Charcot–Marie‐Tooth neuropathy type 1A	scAAV1.tMCK.NTF3 (Nationwide Children's Hospital)	Intramuscular	NCT03520751 (Phase 1/2)
AAV	AAV9	CLN3	Batten disease	AT‐GTX‐502 (Amicus Therapeutics)	Intrathecal	NCT03770572 (Phase 1/2)
AAV	AAV9	Gigaxonin	Giant axonal neuropathy	scAAv9/JeT‐GAN (National Institute of Neurological Disorders and Stroke)	Intrathecal	NCT02362438 (Phase 1)
AAV	AAV5	HTT	Huntington disease	AMT‐130 (UniQure Biopharma B.V.)	Intrastriatal	NCT04120493 (Phase 1/2)
AAV	AAVrh.8	hHEXA or hHEXB	Tay‐Sachs or Sandhoff disease	AXO‐AAV‐GM2 (Sio Gene Therapies)	Intracisternal/Intrathecal	NCT04669535 (Phase 1)
AAV	AAV9	CLN6	Variant late‐infantile neuronal ceroid lipofuscinosis	N/A (Medical University of South Carolina)	Intrathecal	NCT02725580 (Phase 1/2)
AAV	AAV2	GDNF	Multiple system atrophy	AT‐GTX‐501 (Amicus Therapeutics)	Putamen infusion	NCT04680065 (Phase 1)
Metabolic diseases (*n* = 27)						
AAV	AAV2/6	IDS	Mucopolysaccharidosis type I (MPS I)	SB‐318 (Sangamo Therapeutics)	Intravenous	NCT02702115 (Phase 1/2)
AAV	AAV4D‐C102	hGLA	Fabry disease	4D‐310 (4D Molecular Therapeutics)	Intravenous	NCT04519749 (Phase 1/2)
AAV	AAV8	GAA	Pompe disease	AT845 (Audentes Therapeutics)	Intravenous	NCT04174105 (Phase 1/2)
AAV	AAV5	PAH	Phenylketonuria (PKU)	BMN 307 (BioMarin Pharmaceutical)	Intravenous	NCT04480567 (Phase 1/2)
AAV	AAV8	hUGT1A1	Crigler‐Najjar syndrome	GNT0003 (Genethon)	Intravenous	NCT03466463 (Phase 1)
AAV	AAV9	LAMP2B	Danon disease	RP‐A501 (Rocket Pharmaceuticals Inc.)	Intravenous	NCT03882437 (Phase 1)
AAV	AAV8	G6Pase	Glycogen storage disease type IA	DTX401 (Ultragenyx Pharmaceutical Inc.)	Intravenous	NCT03517085 (Phase 1/2)
AAV	AAV8	OTC	Ornithine transcarbamylase (OTC) deficiency	DTX301 (Ultragenyx Pharmaceutical Inc.)	Intravenous	NCT02991144 (Phase 1/2)
AAV	Not specified	ATP7B	Wilson's disease	VTX‐801 (Vivet Therapeutics SAS)	Intravenous	NCT04537377 (Phase 1/2)
Hematological diseases (*n* = 26)						
AAV	AAV5	Factor VIII	Hemophilia A	Valoctocogene Roxaparvovec (BioMarin Pharmaceutical)	Intravenous	NCT04323098 (Phase 3)
AAV	AAV2/6	Factor VIII	Hemophilia A	PF‐07055480 (Pfizer)	Intravenous	NCT03061201 (Phase 2)
AAV	AAV Spark10	Factor IX	Hemophilia B	PF‐06838435 (Pfizer)	Intravenous	NCT03861273 (Phase 3)
AAV	AAV5	Factor IX	Hemophilia B	AMT‐061 (UniQure Biopharma B.V.)	Intravenous	NCT02396342 (Phase 1/2)
Musculoskeletal diseases (*n* = 10)						
AAV	AAV9	Microdystrophin	Duchenne muscular dystrophy	SGT‐001 (Solid Biosciences, LLC)	Intravenous	NCT03368742 (Phase 1/2)
AAV	AAV8	hMTM1	X‐Linked myotubular myopathy	AT132 (Audentes Therapeutics)	Intravenous	NCT03199469 (Phase 1/2)
AAV	AAV.rh74	β‐Sarcoglycan	Limb‐Girdle muscular dystrophy, type 2E	SRP‐9003 (Sarepta Therapeutics, Inc.)	Intravenous	NCT03652259 (Phase 1/2)
Others (*n* = 5)						
AAV	Unknwn	hTERT	Critical limb ischemia	AAV‐hTERT (Libella Gene Therapeutics)	Intravenous	NCT04110964 (Phase 1)
AAV	AAV1	SERCA2a	Congestive heart failure	SRD‐001 (Sardocor Corp.)	Intracoronary	NCT04703842 (Phase 1/2)
AAV	AAV2	Human Aquaporin‐1	Radiation induced xerostomia or salivary hypofunction	AAV2hAQP1 (MeiraGTx UK II Ltd)	Intraparotidal	NCT02446249 (Phase 1)
AAV	AAV8	VRC07 human monoclonal antibody	HIV‐1 infections with controlled viremia	AAV8‐VRC07) (National Institute of Allergy and Infectious Diseases)	Intramuscular	NCT03374202 (Phase 1)
AAV	Unknown	hTERT	Aging	AAV‐hTERT (Libella Gene Therapeutics)	Intravenous	NCT04133649 (Phase 1)

Abbreviations: Genes of Interest: AADC, aromatic L‐amino acid decarboxylase; ATP7B, Wilson disease protein; CLN6, ceroid‐lipofuscinosis neuronal protein 6; CNGA3, cyclic nucleotide gated channel subunit alpha 3; G6Pase, glucose 6‐phosphatase; GAA, alpha glucosidase; GBA1, glucocerebrosidase; GLB1, galactosidase beta 1; GDNF, glial cell‐derived neurotrophic factor; hAPOE2, human apolipoprotein E; hCYP4V2, human CYP4V2; hGALC, human galactosylceramidase; hGLA, human galactosidase alpha; hHEXA, human hexosaminidase subunit alpha; hHEXB, human hexosaminidase subunit beta; hMTM1, human myotubularin 1; hRS1, human retinoschisin 1; hSMN, human survival motor neuron; hTERT, human telomerase reverse transcriptase; HTT, huntingtin; hUGT1A1, UDP‐glucuronosyltransferase 1‐1; IDS, iduronate 2‐sulfatase; ND4, NADH–ubiquinone oxidoreductase chain 4; LAMP2B, lysosome‐associated membrane protein 2; NTF3, neurotrophin 3; PAH, phenylalanine hydroxylase; OTC, ornithine transcarbamylase;REP1, Rab escort protein 1; RPGR, retinitis Pigmentosa GTPase Regulator; VEGF, vascular endothelial growth factor; sCD59, soluble CD59; SERCA2a, sarcoplasmic/endoplasmic reticulum Ca^2+^ ATPase 2a.

Nearly one third (29.4%) of the active trials are investigated for treating ophthalmological diseases (Figure 3c and Table 2). Of note, in all of these trials, AAV gene therapies are administered directly into the eye using a localized method such as subretinal and intravitreal injections. This localized administration enables efficient delivery of viral‐vectors to target retinal compartments and also mitigates off‐target effects. More than nine types of ophthalmological diseases are currently studied (Table 2) and a detailed analysis of our search results, by disease indication, is shown in Figure S1c. Notably, retinitis pigmentosa is the most studied ophthalmological indication in the clinic. In these retinitis pigmentosa‐related trials, a functional gene was delivered to retinal cells to replace the mutated or lost copy to restore vision. Depending on the specific type of retinitis pigmentosa, various genes were used in these trials, such as genes encoding channelrhodopsin, Phosphodiesterase 6A, and retinitis pigmentosa GTPase regulator. It should be noted that the mechanism involved in the age‐related macular degeneration trials is different from that in other indications. Instead of replacing/restoring a gene, these trials for treating age‐related macular degeneration use AAV vectors to express a therapeutic protein such as anti‐VEGF Fab.

Other than ophthalmological diseases, neurological, metabolic, hematological, and musculoskeletal diseases are four additional major indications that the current AAV trials are focused on (Figure 3c and Table 2). AAVs are used to treat many diseases within these categories in the clinic (Figure 3c). A detailed breakdown of our search results, by diseases indications, can be seen in Figure S1c. It should be noted that in the case of metabolic diseases, all the identified trials are intended to introduce a gene to express a lost enzyme for treating the diseases. Majority (88.9%) of the trials use liver as the target organ while a few target CNS organs or muscle. In the context of hematological diseases, all the identified trials are investigating two specific diseases, hemophilia A and hemophilia B (Figure S1c). In all of these trials, AAV gene therapies are administered intravenously and use liver as the target organ. A specific attention should be noted that hemophilia is one of the very first areas that AAV‐based gene therapies have been studied for; their safety profile has been demonstrated through many years of development.^89^, ^90^ So, it is not surprising that many candidate products, for example, valoctocogene roxaparvovec, are being investigated in late‐stage (Phase 3) trials.

Clinical success of AAV‐based gene therapies for treating monogenetic diseases catalyzed the exploration of its potential in treating other diseases. Clinical trials using AAV‐based gene therapies for treating diseases other than monogenic disorders are emerging and new indications include cardiovascular diseases, infectious diseases, and even aging (Table 2). For example, AAV gene therapies introducing the expression of a therapeutic protein, VRC07 human monoclonal antibody, are studied in the clinic for treating HIV‐1 Infections. These examples are expanding the therapeutic horizon of AAV gene therapies beyond monogenic diseases.

### 4.2 Adenovirus vector‐based clinical trials

**FIGURE 4:**
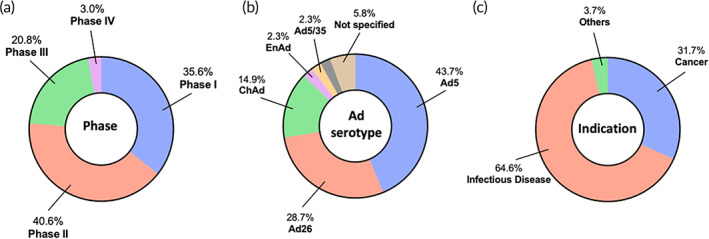
Landscape of adenovirus‐based in vivo gene therapy clinical trials. A total of 83 active clinical trials were identified and further analyzed according to (a) phase, (b) serotype, and (c) indication

Ad vector‐based gene therapy remains one of the most active areas in clinical studies. We identified 83 active clinical trials, most of which are in Phase 1 or 2. However, 24% of the trials have reached Phase 3 or 4 (Figure 4a). This is not surprising since Ad vector‐based gene therapies have historically demonstrated high safety and efficacy.^91^ These identified trials involve the use of various Ad serotypes/variants, with Ad5 and Ad26 being the most widely used (Figure 4b). Other notable clinically investigated serotypes/variants include enadenotucirev (EnAd), Ad VCN‐01, Ad5/35, chimpanzee Ad (ChAd), to name a few (Figure 4b and Table 3). Notably, ChAd is emerging as an important variant in the ongoing clinical trials, most likely attributed to the factor that ChAd is not infectious to humans and thus has a good safety profile.^92^, ^93^ Infectious diseases and cancer are two major indications that the current active trials are focused on (Figure 4c). New areas of indications such as cardiovascular diseases and degenerative diseases are emerging; however, the number of ongoing clinical trials in these areas are only a few (Table 3).

**TABLE 3:** Examples of current clinical trials for adenovirus‐based in vivo gene therapies, grouped by indication

Viral vector	Serotype	Gene of interest	Indication	Name (Sponsor)	Route	Trial number
Cancer (*n* = 26)						
Ad	Ad5	p53	Solid tumor	Ad‐p53 (MultiVir, Inc.)	Intratumoral	NCT03544723 (Phase 2)
Ad	Ad5	N/A	Hepatocellular carcinoma	H101 (Sun Yat‐sen University)	Intravenous	NCT03780049 (Phase 3)
Ad	Ad5	N/A	Refractory malignant ascites	H101 (Fudan University)	Intravenous	NCT04771676 (Phase 2)
Ad	EnAd	Anti‐CD40 antibody	Metastatic cancer, epithelial tumor	NG‐350A (PsiOxus Therapeutics Ltd)	Intratumoral	NCT03852511 (Phase 1)
Ad	Ad VCN‐01	RB1	Refractory retinoblastoma	VCN‐01 (Fundació Sant Joan de Déu)	Intravitreal	NCT03284268 (Phase 1)
Ad	Ad5	IL‐12	Glioblastoma	Ad‐RTS‐hIL‐12 (Ziopharm)	Intratumoral	NCT02026271 (Phase 1)
Ad	Ad5/35	TMZ‐CD40L and 4‐1BBL	Pancreatic cancer	Delolimogene mupadenorepvec (Lokon Pharma AB)	Intratumoral	NCT02705196 (Phase 1/2)
Ad	Ad5	HSV‐tk	Prostate cancer	ADV/HSV‐tk (The Methodist Hospital System)	Intratumoral	NCT03541928 (Phase 2)
Ad	Not specified	IL‐12	Triple negative breast cancer	N/A (The Methodist Hospital System)	Intratumoral	NCT04095689 (Phase 2)
Ad	Ad5	HSV‐tk	Non‐small cell lung cancer	Aglatimagene besadenovec (Candel Therapeutics, Inc.)	Intratumoral	NCT04495153 (Phase 2)
Ad	Ad5	IFNγ	Basal cell carcinoma	ASN‐002 (Ascend Biopharmaceuticals Ltd)	Intratumoral	NCT04416516 (Phase 2)
Ad	Ad5	Fas‐TNFR1	Glioblastoma	VB‐111 (Dana‐Farber Cancer Institute)	Intravenous	NCT04406272 (Phase 2)
Ad	Ad5	yCD, mutTKSR39rep‐ADP	Pancreas cancer	Ad5‐yCD/mutTKSR39rep‐ADP (Seoul National University Bundang Hospital)	Intratumoral	NCT04739046 (Phase 2)
Infectious diseases (*n* = 53)						
Ad	Ad26	Ebola envelope glycoprotein	Ebola virus disease	Ad26.ZEBOV	Intramuscular	NCT04152486 (Phase 3)
Ad	ChAd3	Ebola virus glycoprotein	Ebola virus disease	ChAd3‐EBO Z, VSVG‐ZEBOV (National Institute of Allergy and Infectious Diseases)	Intramuscular	NCT02344407 (Phase 2)
Ad	Ad26	Spike protein	COVID‐19	Ad26.COV2.S	Intramuscular	NCT04838795 (Phase 3)
Ad	Ad26, Ad5	Spike protein	COVID‐19	Gam‐COVID‐Vac	Intramuscular	NCT04564716 (Phase 3)
Ad	Ad5	Spike protein	COVID‐19	Ad5‐nCoV (CanSino Biologics Inc.)	Intramuscular	NCT04526990 (Phase 3)
Ad	ChAd	Spike protein	COVID‐19	AZD1222	Intramuscular	NCT04516746 (Phase 3)
Ad	Ad	Spike protein	COVID‐19	BBV154 (Bharat Biotech International Limited)	Intramuscular	NCT04751682 (Phase 1)
Ad	ChAd68	Spike protein	COVID‐19	ChAdV68‐S, ChAdV68‐S‐TCE (National Institute of Allergy and Infectious Diseases)	Intramuscular	NCT04776317 (Phase 1)
Ad	Ad26, Ad5	Envelope protein	MERS	BVRS‐GamVac (Gamaleya Research Institute of Epidemiology and Microbiology)	Intramuscular	NCT04130594 (Phase 1/2)
Ad	Ad26	Evn sequence, Gag and Pol sequence	HIV infection	Ad26.Mos.HIV, MVA‐Mosaic (Janssen Vaccines & Prevention B.V.)	Intramuscular	NCT02315703 (Phase 1/2)
Ad	ChAd	Fusion HBc and HBs antigen	Hepatitis B infection, chronic	ChAd155‐hIi‐HBV (GlaxoSmithKline)	Intramuscular	NCT03866187 (Phase 1)
Ad	ChAdY25	M.tb antigen 85A	TB infection	ChAdOx185A, MVA85A (University of Oxford)	Intramuscular	NCT03681860 (Phase 1/2)
Others (*n* = 3)						
Ad	Ad5	IL‐1Ra	Knee osteoarthritis	FX201, aka humantakinogene hadenovec (Flexion Therapeutics, Inc.)	Intra‐articular	NCT04119687 (Phase 1)
Ad	Not specified	VEGF	Coronary artery disease	XC001 (XyloCor Therapeutics, Inc.)	Transthoracic epicardial	NCT04125732 (Phase 1/2)
Ad	Not specified	VEGF	Refractory angina pectoris	AdvVEGF‐D (Kuopio University Hospital)	Intramyocardial	NCT03039751 (Phase 1/2)

Abbreviations: Serotypes: ChAd, chimpanzee adenovirus; EnAd, enadenotucirev. Genes of Interest: 4‐1BB ligand, 4‐1BBL; IL‐12, interleukin; HSV‐tk, herpes simplex virus thymidine kinase; IL‐1Ra, interleukin‐1 receptor antagonist; RB1, RB Transcriptional Corepressor 1; VEGF, vascular endothelial growth factor. Indication: HIV, human immunodeficiency virus; MERS, Middle East Respiratory Syndrome; TB, tuberculosis.

Ongoing clinical trials using Ad vector‐based gene therapies for treating infectious diseases are abundant (Table 3) and it should be noted that all these infectious disease‐related trials employ a vaccination mechanism. As mentioned in the previous context, distinguishing features of Ad vectors, including transient but high gene expression and high immunogenicity of the vectors, make them ideal candidates for vaccination applications.^29^ COVID19 and Ebola viruses are two dominant specific disease types among the identified infectious disease‐related clinical trials. More than half of these trials are related to approved products including Ad26.ZEBOV for Ebola and JNJ‐78436735, Sputnik V, Ad5‐nCoV, and AZD1222 for COVID19. Further analysis revealed that these ongoing trials are focused on: (1) seeking approval in different countries, (2) investigating their efficacy in specific population groups, (3) studying different dose regimes, (4) comparing the efficacy of one approved product to that of another, and (5) investigating the combination of one product with another. Other than COVID19 and Ebola virus infection, a number of additional infectious diseases are also being studied in the identified ongoing trials, including Middle East Respiratory Syndrome (MERS), HIV, Hepatitis B, and tuberculosis (TB). The clinical success of Ebola and COVID19 vaccines, especially COVID19 vaccines, will undoubtedly motivate future clinical studies of Ad‐based vaccines. We anticipate that more Ad‐based vaccine clinical trials for a broad array of infectious diseases will continue to emerge.

Cancer is the second most investigated area for the identified Ad vector‐based gene therapy clinical trials (Table 3). Interestingly, although two Ad vector‐based products have been approved, their current investigation in ongoing trials is minimal; two Oncorine® and two Gendicine® related trials are studying their application in additional cancer types. For example, Oncorine® is investigated for treating hepatocellular carcinoma and refractory malignant ascites. Majority of the cancer‐related clinical trials are studying newer candidates for managing various types of cancer including pancreas cancer, glioblastoma, lung cancer, basal cell carcinoma, breast cancer, prostate cancer, to name a few. Many potent protein therapeutics, such as immunomodulatory cytokines and antibodies, have been identified over the decade for cancer treatments. The newer Ad‐based cancer gene therapy candidates in the active clinical trials are incorporating genes encoding these potent protein therapeutics. Notable genes of interest include anti‐CD40 antibody, IL‐12, TMZ‐CD40L, 4‐1BBL, HSV‐tk, IFN‐γ, and many others (Table 3). It should be noted that in many cases, these newer candidates can involve different modes of anti‐cancer activities (e.g., viral vector‐mediated tumor lysis and protein therapeutic‐induced immunomodulation) for synergistically suppressing tumors.

### 4.3 HSV vector‐based clinical trials

**FIGURE 5:**
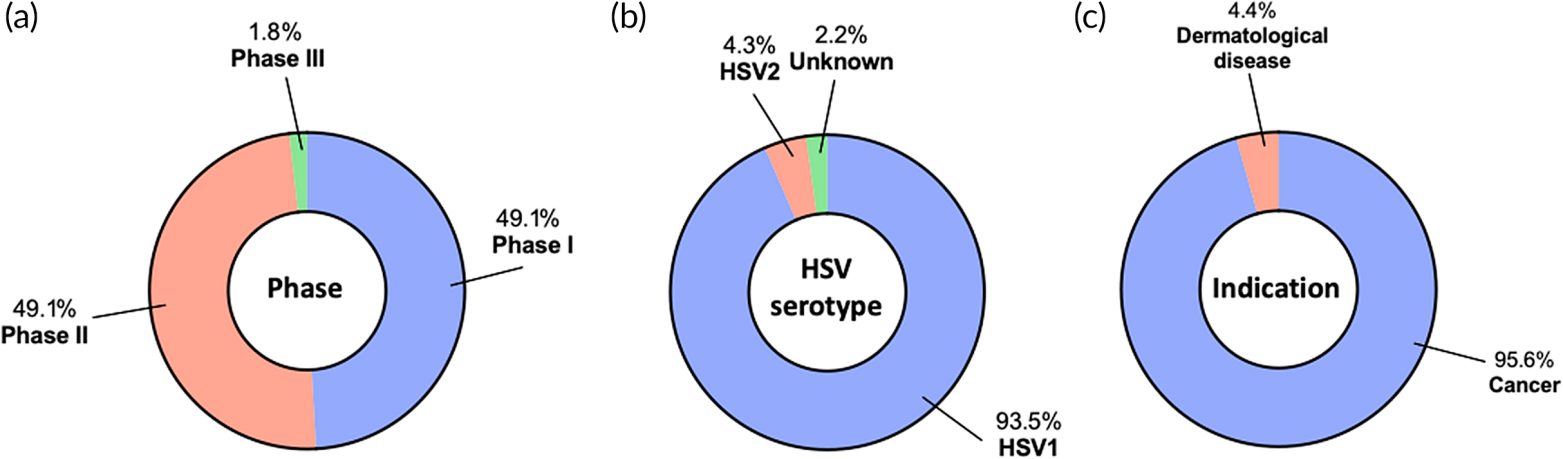
Landscape of herpes simplex virus‐based in vivo gene therapy clinical trials. A total of 46 active clinical trials were identified and further analyzed according to (a) phase, (b) serotype, and (c) indication

US FDA has approved a HSV‐based gene therapy (IMLYGIC®) for cancer applications, so it is not surprising that a large number of clinical trials are investigating HSV‐based gene therapies. We identified 46 unique HSV‐based gene therapy trials. Majority of the identified trials are in early‐stages while one trial reached Phase 3 (Figure 5a). Analysis of HSV serotypes used in these trials revealed that HSV1 is the most used (Figure 5b). This is understandable because the clinical approval of IMLYGIC® proved the outstanding safety profile and promising therapeutic potential of the HSV1 vector.

Almost all of the identified trials focus on cancer applications, most likely attributed to the demonstrated oncolytic property of HSV vectors (Figure 5c). Majority (77.3%) of the cancer trials are concentrated on the approved IMLYGIC® for treating additional types of cancers, such as sarcoma, skin squamous cell carcinoma, breast cancer, ovarian cancer, rectal cancer, pancreatic cancer, and liver cancer (Table 4). Approval for additional indications for an approved product is typically more direct as compared to a new technology, so it is not surprising that so many trials are related to IMLYGIC® for treating additional cancer types. The non‐IMLYGIC®‐related trials for cancer applications are studying other HSV serotypes or newer genes of interest (Table 4). For example, an HSV2‐based candidate is currently in Phase 1/2 trials for treating gastrointestinal cancer and melanoma. IMLYGIC® carries a gene encoding GM‐CSF to immunologically boost its cancer therapeutic efficacy. Genes of interest are expanding beyond GM‐CSF in the non‐IMLYGIC®‐related trials, including genes encoding anti‐CTLA‐4 antibody, CD40 ligand, 4‐1BBL, IL‐12, fusogenic protein, and Thymidine Kinase‐m2 (Table 4).

**TABLE 4:** Examples of current clinical trials for herpes simplex virus (HSV)‐based in vivo gene therapies, grouped by indication

Viral vector	Serotype	Gene of interest	Indication	Name (Sponsor)	Route	Trial number
Cancer (*n* = 44)						
HSV	HSV1	GM‐CSF	Soft tissue sarcoma	Talimogene Laherparepvec (Amgen)	Intratumoral	NCT04599062 (Phase 1/2)
HSV	HSV1	GM‐CSF	Breast cancer	Talimogene Laherparepvec (Honsson Comprehensive Cancer Center)	Intratumoral	NCT04185311 (Phase 1)
HSV	HSV1	GM‐CSF	Melanoma	Talimogene Laherparepvec (Amgen)	Intratumoral	NCT04427306 (Phase 2)
HSV	HSV1	GM‐CSF	Peritoneal surface malignancies	Talimogene Laherparepvec (Duke University)	Intratumoral	NCT03663712 (Phase 2)
HSV	HSV1	GM‐CSF	Triple negative breast cancer and colorectal cancer with liver metastases	Talimogene Laherparepvec (Amgen)	Intratumoral	NCT03256344 (Phase 1)
HSV	HSV1	GM‐CSF	Rectal cancer	Talimogene Laherparepvec (National Cancer Institute)	Intratumoral	NCT03300544 (Phase 1)
HSV	HSV1	GM‐CSF	Pancreatic cancer	Talimogene Laherparepvec (Amgen)	Intratumoral	NCT03086642 (Phase 1)
HSV	HSV1	GM‐CSF	Hepatocellular carcinoma	Talimogene Laherparepvec (Amgen)	Intratumoral	NCT02509507 (Phase 1/2)
HSV	HSV2	GM‐CSF	Solid tumor, gastrointestinal cancer	OH2 (Wuhan Binhui Biotechnology Co., Ltd.)	Intratumoral	NCT03866525 (Phase 1/2)
HSV	HSV1	CYP2B1	Liver metastases, primary liver cancers	rRp450 (Massachusetts General Hospital)	Intra‐hepatic artery	NCT01071941 (Phase 1)
HSV	HSV1	Anti‐CTLA‐4 antibody, CD40 ligand, and 4‐1BBL	Advanced solid tumor	RP3 (Replimune Inc.)	Intratumoral	NCT04735978 (Phase 1)
HSV	HSV1	IL‐12	Recurrent malignant glioma	M032 (University of Alabama at Birmingham)	Intratumoral	NCT02062827 (Phase 1)
HSV	HSV1	Fusogenic protein (GALV‐GP‐R^−^) and GM‐CSF	Advanced squamous skin cancer	RP1 (Replimune Inc.)	Intratumoral	NCT04050436 (Phase 2)
HSV	HSV1	Thymidine Kinase‐m2 and GM‐CSF	Hepatocellular carcinoma	GEN2 (GenVivo, Inc.)	Intravenous	NCT04313868 (Phase 1)
Others (*n* = 2)						
HSV	HSV1	TGM1	TGM‐1 related autosomal recessive congenital ichthyosis	KB105 (Krystal Biotech, Inc.)	Topical	NCT04047732 (Phase 1/2)
HSV	HSV1	COL7	Dystrophic epidermolysis bullosa	KB103 (Krystal Biotech, Inc.)	Topical	NCT03536143 (Phase 1)

Abbreviations: Genes of Interest: 4‐1BBL, 4‐1BB ligand; COL7, collagen type VII; GM‐CSF, Granulocyte‐macrophage colony‐stimulating factor; IL‐12, interleukin‐12; TGM1, transglutaminase 1.

Additional indications other than cancer applications are rare in the identified HSV‐based gene therapy clinical trials. However, some new areas are emerging. For example, treatment of dermatological diseases is involved in a few of the identified trials (Table 4). As an example, KB105, a replication‐incompetent, nonintegrating HSV1 vector expressing human transglutaminase 1 (TGM‐1) is currently studied for the management of TGM‐1‐related autosomal recessive congenital ichthyosis.

### 4.4 Other viral vector‐based clinical trials

We identified 18 ongoing clinical trials using newer viral vectors other than AAV, Ad, and HSV for in vivo gene therapies (Table 5). These newer viral vectors include lentivirus, arenavirus, measles virus, MVA, fowlpox virus, VSV, human cytomegalovirus, retrovirus, Sendai virus, among others. Depending on properties of specific viral vectors, various indications using these viral vector‐based gene therapies are emerging. Notable indications include cancer, infectious diseases, and neurological diseases. Of note, lentivirus‐based in vivo gene therapies are emerging as an important area in the active clinical trial landscape. There are already ongoing clinical trials that investigate lentivirus‐based gene therapies for treating cancer, infectious diseases, neurological diseases, metabolic disease, and ophthalmological disease. With better understanding of the biology of these newer viral vectors, it is expected that they will be more extensively examined in clinical trials for various indications.

**TABLE 5:** Examples of current clinical trials for other viral vector‐based in vivo gene therapies, grouped by indication

Viral vector	Serotype	Gene of interest	Indication	Name (Sponsor)	Route	Trial number
Cancer (*n* = 6)						
Arenavirus	N/A	E6/E7 fusion protein	HPV‐related squamous cell carcinoma	HB‐201, HB‐202 (Hookipa Biotech GmbH)	Intravenous	NCT04180215 (Phase 1/2)
Measles Virus	N/A	*Helicobacter pylori* Neutrophil‐activating Protein	Metastatic breast cancer	MV‐s‐NAP (Mayo Clinic)	Intratumoral	NCT04521764 (Phase 1)
MVA	N/A	p53	Solid tumors	MVA‐p53 (City of Hope Medical Center)	Subcutaneous	NCT02432963 (Phase 1)
Fowlpox viral vector, vaccinia virus	N/A	CEA, MUC‐1	Pancreas cancer	Falimarev, Inalimarev (National Cancer Institute)	Intratumoral	NCT00669734 (Phase 1)
Vesicular Stomatitis Virus	N/A	Interferon‐beta	Refractory liver cancer or advanced solid tumors	VSV‐hIFN‐b (Mayo Clinic)	Intratumoral	NCT01628640 (Phase 1)
Infectious diseases (*n* = 6)						
Lentivirus	N/A	HCV antigens	Chronic hepatitis C infection	HCVax (GeneCure Biotechnologies)	Intravenous	NCT04318379 (Phase 1)
Human cytomegalovirus	N/A		HIV I infection	VIR‐1111 (Vir Biotechnology, Inc.)	Subcutaneous	NCT04725877 (Phase 1)
Measles Virus	N/A	Surface glycoprotein	COVID19	TMV‐083 (Institut Pasteur)	Intramuscular	NCT04497298 (Phase 1)
Retrovirus	N/A	cytocidal dominant negative human cyclin G1	COVID19	DeltaRex‐G (Aveni Foundation)	Intravenous	NCT04378244 (Phase 1)
MVA	N/A	Spike protein	MERS	MVA‐MERS‐S_DF1 (Universitätsklinikum Hamburg‐Eppendorf)	Intramuscular	NCT04119440 (Phase 1)
MVA	N/A	M3 and M4 antigen	HIV‐1 infection	MVA.tHIVconsv3 (University of North Carolina at Chapel Hill)	Intramuscular	NCT03844386 (Phase 1)
Neurological diseases (*n* = 3)						
Lentivirus	N/A	Potassium channel	Epilepsy	N/A (University College London)	Intracranial	NCT04601974 (Phase 1/2)
Lentivirus	N/A	TH, AADC, CH1	Parkinson's disease	OXB‐102 (Sio Gene Therapies)	Neurosurgical	NCT03720418 (Phase 1/2)
Lentivirus	N/A	ABCD1	X‐linked adrenoleukodystrophy	N/A (Shenzhen Geno‐Immune Medical Institute)	Intracerebral	NCT03727555 (Phase 1)
Others (*n* = 3)						
Lentivirus	N/A	ARSA	Metachromatic leukodystrophy	N/A (Shenzhen Geno‐Immune Medical Institute)	Intracerebral	NCT03725670 (Phase 1)
Lentivirus	N/A	Endostatin and Angiostatin	Age‐related macular degeneration	RetinoStat (Oxford BioMedica)	Subretinal	NCT01678872 (Phase 1)
Sendai virus	N/A	hFGF‐2	Intermittent claudication, Peripheral arterial disease	DVC1‐0101 (Kyushu University)	Intramuscular	NCT02276937 (Phase 2)

Abbreviations: Viral Vector: MVA, Modified Vaccinia virus Ankara. Genes of Interest: AADC, aromatic l‐amino acid decarboxylase, ABCD1, peroxisomal ATP‐binding cassette transporter; ARSA, arylsulfatase A; CEA, carcinoembryonic antigen; CH1, GTP‐cyclohydrolase; hFGF‐2, human fibroblast growth factor‐2; MUC1, Mucin 1; TH, tyrosine hydroxylase. Indications: HIV, human immunodeficiency virus; MERS, Middle East Respiratory Syndrome.

## 5 CLINICAL CHALLENGES

Viral vector‐based gene therapies face many challenges that can affect their implementation and adoption into standard clinical care; indeed, these challenges have been extensively reviewed in previous articles.^94^, ^95^, ^96^, ^97^, ^98^, ^99^, ^100^, ^101^, ^102^, ^103^ A main challenge is the reliance on animal models that inaccurately predict transduction efficiency in humans; one effort in this area is the development of humanized mouse models or animal models that use tissue grafts from humans to better predict potential efficacy and success of viral vector‐based gene therapies in the clinic.^42^ Closely connected to efficacy in humans, immune response from viral vectors presents a significant hurdle to the translation of viral vector‐based gene therapies. Briefly, both innate and adaptive immune responses against viral vector‐based gene therapies can occur in human patients, which can limit: (1) repeated use of the same viral vector due to host‐generation of neutralizing antibodies, which can alter biodistribution and thus prevent the successful long‐term use of viral vectors, and (2) transduction efficiency due to immune system response to the introduced viral structures.^94^ Importantly, understanding how to navigate and interact with the immune system, and possibly control immune response, could lead to the expansion of applications that viral vector‐based gene therapies could be clinically used for. Approaches to mitigate immune responses against viral vectors include introduction of novel vectors with tissue‐specific tropism,^104^ genetically engineering the capsid through directed evolution,^42^ and altering the route of administration,^105^ to name a few currently emerging approaches.^106^, ^107^, ^108^ There also exists considerable manufacturing challenges, which have been previously and extensively reviewed^95^, ^96^, ^97^, ^101^, ^102^, ^103^; importantly, these limitations with manufacturing and productions can contribute to the high costs of many viral vector‐based gene therapies, especially those for rare diseases.^98^ To address these production and manufacturing challenges, emerging efforts include transition from transfected producer cells to stable producer cell lines,^95^ development of processes that enable suspension culture (as opposed to adherent‐culture) of producer cell lines,^96^ development of serotype‐independent purification processes for viral vectors,^109^ and new methodologies to identify contaminating proteins or bacteria of vector stocks,^102^ to name a few. Through our search, we identified greater than 20 recent viral vector‐based clinical trials that had been withdrawn or terminated; however, reasons for the withdrawal or termination for the overwhelming majority of these trials were either not stated or provided generic reasons (e.g., sponsor decision, low accrual). Two studies provided specific reasons for trial termination. NCT02387125, a clinical trial studying the lentiviral vector CMB305 to target dendritic cells as a prime‐boost vaccine against tumors that express NY‐ESO‐1, was terminated on July 1, 2020 due to the clinical trial not meeting the efficacy objective. Likewise, NCT02609984, which also studied CMB305 was terminated on July 7, 2020, also due to lack of meeting the efficacy objective. While specific reasons for lack of efficacy were not listed, the results for the latter study have been posted on clinicaltrials.gov; overall, these studies highlight the difficulty in achieving efficacy for viral vectors, potentially due to the aforementioned clinical challenges.

## 6 CONCLUSIONS

Gene therapy directly corrects/modifies genes and represents a revolutionary modality to treat diseases. Gene therapy holds a unique position in the therapeutics spectrum in a sense that it is the only approach that can potentially cure some diseases such as genetic disorders. Many gene therapy products, viral vector‐based gene therapies in particular, have received approval in the clinic for managing a broad array of diseases including not only genetic disorders but other indications such as cancer and infectious diseases. Particularly, recent approvals of AAV‐based products have revolutionized the field and offered, for the first time, a therapy to cure monogenetic diseases. Beyond that, a number of active clinical trials are emerging in the clinical landscape investigating newer viral vector‐based gene therapies. These ongoing clinical efforts are studying newer types of viral vectors, incorporating novel gene modification approaches (e.g. CRISPR‐mediated gene editing), and more importantly, expanding gene therapy's therapeutic spectrum toward new indications. Yet, further clinical translation of viral gene therapies is facing manufacturing, biological, and regulatory challenges that requires significant efforts from the preclinical, clinical, and commercial sides. However, scientific advances in the area of viral vector engineering, disease genomic identification, and base‐level gene editing are bring viral gene therapies to a new era. Viral vector‐based gene therapy is expected to remain a highly active area in the clinic and more gene therapy products are expected to be seen in the market.

## Supporting information


**Table S1** Abbreviation list.
**Figure S1.** Analysis of AAV‐based in vivo gene therapy clinical trials. Current active clinical trials were analyzed according to a) administration method and b) tissue target. c) Number of active clinical trials for specific diseases within different indication categories.
