# Supplementary material for: Viral vector‐based gene therapies in the clinic
Source: Bioeng Transl Med. 2021 Oct 20;7(1):e10258. doi: 10.1002/btm2.10258 (PMC8780015; doi:10.1002/btm2.10258)
Supplement: Supplementary file 1 — Table S1 Abbreviation list. Figure S1. Analysis of AAV‐based in vivo gene therapy clinical trials. Current active clinical trials were analyzed according to a) administration method and b) tissue target. c) Number of active clinical trials for specific diseases within different indication categories. [file BTM2-7-e10258-s001.docx]

Supplementary Information

**Viral Vector-Based Gene Therapies in the Clinic**

Zongmin Zhao^1,*^, Aaron C. Anselmo^2,*^, Samir Mitragotri^3,4,*^

1. Department of Pharmaceutical Sciences, College of Pharmacy, University of Illinois at Chicago, Chicago, IL 60612, USA

2. Division of Pharmacoengineering and Molecular Pharmaceutics, Eshelman School of Pharmacy, University of North Carolina at Chapel Hill, Chapel Hill, NC 27599, USA

3. John A. Paulson School of Engineering and Applied Sciences, Harvard University, Cambridge, MA 02138, USA

4. Wyss Institute for Biologically Inspired Engineering at Harvard University, Boston, MA 02115, USA

^*^Corresponding author

Email: [zhaozm@uic.edu](mailto:zhaozm@uic.edu)

Email: [aanselmo@email.unc.edu](mailto:aanselmo@email.unc.edu)

Email: [mitragotri@seas.harvard.edu](mailto:mitragotri@seas.harvard.edu)

**Supplementary Table 1.** Abbreviation list.

| Abbreviation | Full Name |
| --- | --- |
| SCID | severe combined immunodeficiency |
| AAV | adeno-associated virus |
| Ad | adenovirus |
| cDNA | Complementary DNA |
| ChAd | chimpanzee adenovirus |
| CNS | Central nervous system |
| Cryo-EM | cryogenic electron microscopy |
| CTLA-4 | cytotoxic T-lymphocyte-associated protein 4 |
| EMA | European Medicines Agency |
| Fab | fragment, antigen-binding |
| HSV | herpes simplex virus |
| GM-CSF | granulocyte macrophage colony-stimulating factor |
| HIV | human immunodeficiency virus |
| HNSCC | head and neck squamous cell carcinoma |
| ICP | infected cell protein |
| IL-12 | interleukin-12 |
| MERS | Middle East Respiratory Syndrome |
| MVA | modified vaccinia virus Ankara |
| TB | tuberculosis |
| TGM-1 | transglutaminase-1 |
| VEGF | vascular endothelial growth factor |
| VSV | vesicular stomatitis virus |
| 4-1BBL | 4-1BB ligand |


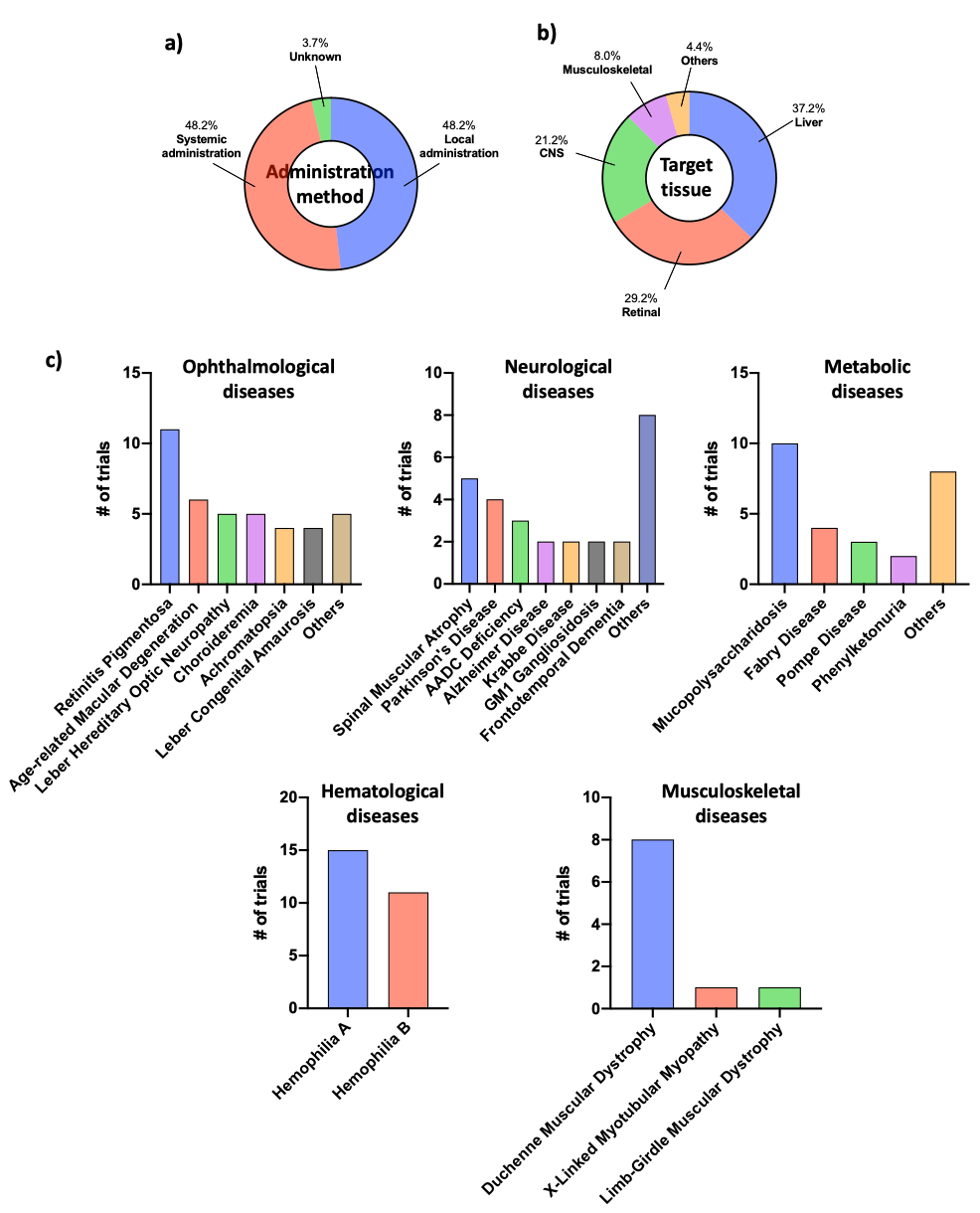


**Figure S1. Analysis of AAV-based *in vivo* gene therapy clinical trials.** Current active clinical trials were analyzed according to a) administration method and b) tissue target. c) Number of active clinical trials for specific diseases within different indication categories.
